# Assessing the enzymatic effects of cellulases and LPMO in improving mechanical fibrillation of cotton linters

**DOI:** 10.1186/s13068-019-1502-z

**Published:** 2019-06-26

**Authors:** Cristina Valls, F. I. Javier Pastor, M. Blanca Roncero, Teresa Vidal, Pilar Diaz, Josefina Martínez, Susana V. Valenzuela

**Affiliations:** 1 Department of Genetics, Microbiology and Statistics, Faculty of Biology, Universitat de Barcelona, Av. Diagonal 643, 08028 Barcelona, Spain; 2 CELBIOTECH_Paper Engineering Research Group, Universitat Politècnica de Catalunya, BarcelonaTech, 08222 Terrassa, Spain

**Keywords:** Cellulose, Cotton linters, LPMO, Laccase_Tempo, Cellulases, NFC

## Abstract

**Background:**

The increasing interest in replacing petroleum-based products by more sustainable materials in the packaging sector gives relevance to cellulose as a biodegradable natural resource. Moreover, its properties can be modified physically, chemically or biotechnologically in order to obtain new bioproducts. Refined cotton linters with high cellulose content were treated with hydrolytic (cellulases) and oxidative (LPMO and Laccase_Tempo) enzymes to evaluate their effect on fibre properties and in improving mechanical fibrillation.

**Results:**

Cellulases released cellooligosaccharides, reducing fibre length and partially degrading cellulose. They also improved mechanical fibrillation yielding up to 18% of nanofibrillated cellulose (NFC). LPMO introduced a slight amount of COOH groups in cellulose fibres, releasing cellobionic acid to the effluents. The action of cellulases was improved after LPMO treatment; however, the COOH groups created disappeared from fibres. After mechanical fibrillation of LPMO–cellulase-treated cotton linters a 23% yield of NFC was obtained. Laccase_Tempo treatment also introduced COOH groups in cellulose fibres from cotton, yielding 10% of NFC. Degree of polymerization was reduced by Laccase_Tempo, while LPMO treatment did not significantly affect it but produced a higher reduction in fibre length. The combined treatment with LPMO and cellulase provided films with higher transparency (86%), crystallinity (92%), smoothness and improved barrier properties to air and water than films casted from non-treated linters and from commercial NFC.

**Conclusions:**

The combined enzymatic treatment with LPMO and cellulases boosted mechanical fibrillation of cotton linters, improving the NFC production and providing bioproducts with high transparency and high barrier properties.

**Electronic supplementary material:**

The online version of this article (10.1186/s13068-019-1502-z) contains supplementary material, which is available to authorized users.

## Background

Cotton linters are an important by-product of the textile industry, being the short fibre fraction that cannot be used in the textile process [1]. They are obtained from cotton plant (*Gossypium* sp.), an annual shrub harvested for their high industrial interest. Cotton linters consist of high-quality cellulose fibres presenting very high cellulose content (98%) [2]. They are typically used in special applications such as the production of cellulose derivatives, regenerated cellulose, or the manufacture of high added-value papers [3].

In order to construct new materials and products based on renewable resources, the interest in functionalizing cellulose has gained importance in the last years. In fact, there is an increasing interest in replacing synthetic polymers by more sustainable materials to replace petroleum-based products in the packaging sector [4]. Modification of cellulose by chemical or biotechnological means has been reported [5]. Hydrolytic enzymes like cellulases can successfully modify cellulose, improving its reactivity and also altering fibre morphology [6]. On the other hand, the oxidative enzymatic system Laccase_Tempo (2,2,6,6-tetramethyl-1-piperidinyloxy) can create new functional groups to cellulose converting primary hydroxyl groups to aldehyde or carboxyl forms [7, 8]. In this system, laccase, having a redox potential in the range of 0.7–0.9 V, can easily oxidize the stable oxyl-radical form of Tempo to oxoammonium ion (*E*° 0.2 V). This ion is the actual oxidant of cellulose, which can be regenerated by laccase oxidation or by acid-induced disproportionation.

A new generation of enzymes that also create functional groups in cellulose and other crystalline polysaccharides such as chitin, lytic polysaccharide monooxygenases (LPMO), have been discovered [9, 10]. They oxidatively cleave glycosidic linkages, leading to the formation of oxidized glucose units at different positions, resulting in the formation of aldonic acids at the C1 position and/or 4-ketoaldoses (gemdiols) at the C4 position [11]. This oxidation renders the substrate more susceptible to be hydrolysed by conventional cellulases and is considered as a breakthrough in the enzymatic degradation of cellulose [12]. The enzymatic effects that LPMO produce in cellulose have been mainly evaluated through their increase in cellulose degradation [13, 14]. However, the effect that LPMO produces on pulp fibres has been poorly investigated [15–17]. These authors demonstrated that LPMO weakens fibres cohesion, promoting their disruption during mechanical fibrillation.

The production of nanocrystalline cellulose (NCC) from cotton linters has been reported, and also the ability of cellulases to improve its yield [2, 18]. However, little knowledge exists about the production of nanofibrillated cellulose (NFC) from these fibres [19–21]. Interestingly, cotton provides fibres with promising interest in nanocellulose production due to its high purity and highly crystalline cellulose [2]. NFC is usually produced by high-pressure homogenization, being major impediments for its commercial success the very high energy consumption of the production process and the clogging of homogenizers. Therefore, some pretreatments are needed in order to facilitate this process [22]. The ability of cellulases to improve this process has been demonstrated [23–26]. Also, the improvement of mechanical fibrillation produced with Tempo–NaBr–NaClO system is well known [20]. In order to replace the halide-based co-oxidizer system, laccase can be used to oxidize Tempo.

In this work, several enzymes were applied on cotton linters in order to analyse their effects on sugar release and on cellulose and fibre modifications. Four hydrolytic enzymes (cellulases) and also two oxidative enzymatic systems (a new bacterial LPMO and the Laccase_Tempo system) were used for this purpose. The effects that enzymes produced during mechanical fibrillation were also analysed, together with the optical, physical and barrier properties of the films casted from these treated linters.

## Materials and methods

### Raw material

Cotton linters obtained from the second cut were supplied by CELESA (Celulosa de Levante S.A.), Tortosa, Spain. The initial fibres had an average of 0.47 mm of length, 19.67 μm of width and 38.87% fine content. Their drainability, measured as ºSR, was 12. Prior to the enzymatic pretreatments, cotton linters were refined in a valley mill for 24 h in order to reduce their average length. Refined linters, named as “R”, had an average fibre length of 0.25 mm, fibre with 25.5 μm and 52.58% of fines; their drainability was increased to 77ºSR.

A commercial NFC (Com) supplied by the University of Maine, with 90% of fines, was used for comparison.

### Enzymes

Four hydrolytic enzymes (cellulases) and two oxidative enzymes (LPMO and Laccase_Tempo) were used as pretreatments in cotton linters. Cel9B from *Paenibacillus barcinonensis* BP-23 [27] was a monocomponent processive endoglucanase named as “C_9_”. A commercial cellulase from Sertec20 was named as “C_50_”, whereas two commercial cellulases supplied by Novozymes^®^ (Fibercare and Celluclast) were named as “C_F_” and “C_ll_”, respectively. Their initial enzymatic activities were 5.5, 383, 99 and 536 U mL^−1^ for C_9_, C_50_, C_F_ and C_ll_, respectively. The commercial cellulase preparations used were not monocomponent and contained mixtures of several enzymes. Enzymatic activity was assayed by measuring the amount of reducing sugars released from carboxymethylcellulose (CMC) by the dinitrosalicylic (DNS) reagent method [28]. The standard assay (100 µL reaction volume) was performed at 50 °C in 50 mM potassium acetate buffer at pH 5 for 15 min. One unit of enzymatic activity (U) was defined as the amount of enzyme that releases 1 µmol of reducing sugar equivalent per min under the assay conditions described. A standard curve of glucose was used to calculate activity units. All determinations of enzyme activity were made in triplicate.

For the oxidative treatments, an LPMO from *Streptomyces ambofaciens* (SamLPMO10C) [29] and a laccase from *Trametes villosa* in combination with Tempo (2,2,6,6-tetramethyl-1-piperidinyloxy) were used. They were named as “S” and “L_Tempo”, respectively. Laccase was supplied by Novozymes^®^ (Denmark) and had an activity of 746 U mL^−1^. Tempo was purchased from Sigma-Aldrich. The laccase activity was measured as the extent of oxidation of 5 mM 2,20-azino-bis(3-ethylbenzothiazoline-6-sulphonic acid) (ABTS) to its cation radical (*ε*_436_ = 29,300 M^−1^ cm^−1^) in 0.1 M sodium acetate buffer (pH 5) at 24 °C. One activity unit (U) was defined as the amount of enzyme converting 1 µmol of ABTS per min.

### Enzymatic pretreatments on cotton linters

Pretreatments with cellulases were performed with 5 g odp (oven-dried pulp) at 10% consistency, with 10 U g^−1^ odp of enzyme in 50 mM potassium acetate buffer, pH 5, at 50 °C for 18 h. A combined treatment with C_F_ and C_ll_, named as “C_mix_”, was also performed. This pretreatment was performed as described above but with 10 U g^−1^ odp of C_F_ and 10 U g^−1^ odp of C_ll_. Treatment with LPMO (S treatment) was performed with 5 g odp and 4 mg of enzyme g^−1^ odp at 5% consistency, for 72 h at 50 °C in 10 mM of ammonium acetate buffer at pH 6, with 2 mM ascorbic acid and 20 μM hydrogen peroxide. L_Tempo oxidation treatments were performed at room temperature, at 5% consistency, using 50 mM potassium acetate buffer at pH 5, 60 U g^−1^ odp laccase and 8% odp of Tempo for 18 h, according to previous works [7, 8].

All the enzymatic treatments were conducted in polyethylene bags that were placed in a laboratory water bath. After treatment, liquors were recovered and the resulting pulp was extensively washed as reported elsewhere for eucalyptus pulp [30] in order to remove the enzymes and their degradation products. In the case of L_Tempo treatments, pulp was also washed with ethanol. Control treatments with potassium acetate buffer and ammonium acetate buffer were also performed at the same application conditions but without the addition of enzymes. They were named “C_K_” and “S_K_”.

### Effects on effluent properties

Released cellooligosaccharides were quantified by the dinitrosalicylic (DNS) reagent method and analysed by thin-layer chromatography (TLC) and HPAEC-PAD (high-performance anion-exchange chromatography with pulsed amperometric detection). For reducing sugar quantification, 100 μL of DNS was added to 100 μL samples and mixtures were incubated at 100 °C for 5 min. Then, 40 μL of reaction mixtures was placed in ELISA plates, 260 μL of distilled water was added, and the absorbance at 540 nm was measured. Samples were analysed in triplicate. A standard curve of glucose was used to calculate the glucose reducing sugar equivalent of the different samples [31].

For TLC analysis 10–15 μL of samples was applied on a silica gel plate (Merck, Germany) constituting the solid phase. 10 μL of an oligomer standard mixture containing cellooligosaccharides at a concentration of 20 mg mL^−1^ was applied as migration standards. The mobile phase was a mixture of chloroform, acetic acid and H_2_O in 6:7:1 ratio, respectively. The migration was repeated twice, and the silica gel plate was then sprayed (Fungilab S.A., Spain) with a developing solution, consisting of 5% H_2_SO_4_ in ethanol. Finally, the plate was heated in the oven at 100 °C for 5 min, where the spots corresponding to different cellooligosaccharides were visualized [31]. For HPAEC-PAD sample preparation, after removing insoluble substrates by centrifugation, supernatants were centrifuged and diluted in water 1/20 and analysed by HPAEC-PAD using Dionex GS50, gradient pump, Dionex AS50 Autosample and electrochemical detector Waters 2465. In brief, 40-μL samples were injected on a CarboPac PA1 2 × 250 mm analytical column (Dionex). Cellooligosaccharides were eluted at 0.25 mL min^−1^ using a stepwise linear gradient from 100% eluent A (0.1 M NaOH) towards 10% eluent B (0.6 M NaOAc in 0.1 M NaOH) 10 min after injection and to 40% eluent B 15 min after injection, followed by a 5-min exponential gradient to 100% B. The column was reconditioned between each run by running initial conditions for 10 min. Standards were generated using 1, 2, 4 and 8 μg mL^−1^ cellobiose and cellobionic acid [17].

### Pulp characterization

The morphological properties of the fibres (viz. length and width) and the content in fines of the pulp samples were determined in accordance with TAPPI T 271 on a Metso kajaani FS300 fibre analyser. All samples were analysed in duplicate. Viscosity was determined according to ISO 5351:2010. The degree of polymerization (DP) was calculated from the intrinsic viscosity (*Ƞ*), using the equation of (SCAN-CM15:88): DP0.085 = 1.1 × [*Ƞ*]. Carboxyl groups were determined by measuring methylene blue adsorption onto cellulose fibres according to Davidson [32]. To measure aldehyde groups samples were further oxidized with NaClO_2_ for selective conversion of aldehyde groups into carboxyl groups at room temperature for 48 h. The carboxyl content was determined with the above-described method. The carboxyl groups formed by effect of NaClO_2_ oxidation were assumed to derive from aldehyde groups originally present in the pulp. Three measures per sample were performed, and the 95% confidence interval was calculated.

### High-pressure homogenization

Prior to fibrillation, 2 g of oven-dried pulp (odp) at 1% consistency was disintegrated for 1 min at 11,200 rpm with a homogenizer (Homogenizing System UNIDRIVE X1000). Then, samples were diluted until 0.5% consistency and homogenized through the PANDA GEA 2000 homogenizer by 5 passes at 300 bar and 10 passes at 900 bar.

The yield of fibrillation (Eq. 1) was calculated after centrifuging 10 mL of a sample at 0.1% consistency at 2200×*g* for 20 min, removing the supernatant (containing the nanofibrillated fraction) and drying pellet (*C*) at 85 °C until constant weight.

1 $${\text{Yield}} = \left( {1 - \frac{{C \left( {\text{g}} \right)}}{{0.01 {\text{g}}}}} \right) \times 100 \%$$

Transmittance measurements were taken on samples with 0.1% of solid content. The sample was introduced in quartz cuvettes, and the transmittance was obtained with a T92 + UV spectrophotometer (PG instruments) set in the range between 400 and 800 nm. Milli-Q water was used as blank.

Fibre morphology and DP were measured as previously described in pulp samples. Electrophoretic mobility of aqueous suspensions (zeta potential) was determined using a Zetamaster model ZEM (Malvern Instruments, UK). Data were averaged over 10 measurements. All samples were analysed at room temperature.

### Film characterization

After fibrillation, films with a grammage around 45–50 g m^−2^ were obtained by the film casting technique [33]. Their optical and physical–mechanical properties were determined in accordance with the standards in brackets as follows: transparency (22891:2013), apparent density (ISO 534:2005), Bekk smoothness (5627:1995), and dry and wet zero-span index (ISO 15361:2000). Fibres zero-span tensile index was determined in a Zero-span 1000 Pulmac tester. For analysis of wet zero-span index, films were previously soaked in distilled water for 120 s.

Barrier properties to air and water were also analysed. Air permeance was measured with Bekk equipment. Water impermeability was measured by the water drop test (WDT) according to TAPPI standard T835 om-08. The WDT involved placing a drop of deionized water on the surface of paper and recording the time needed for complete absorption, which was signalled by vanishing of the drop specular gloss. Ten measurements per treated film sample were made and averaged. Six measures per sample were performed, and the 95% confidence interval was calculated.

The crystallinity index (CrI) of different cellulosic substrates was measured by XRD (X-ray powder diffraction). Samples were dried directly on an aluminium plate of 32 mm of diameter and 3.0 mm of thickness, that were mounted in standard sample holders for bulk samples of thickness ≤ 7 mm (PW1812/00) by means of plasticine. A PANalytical X’Pert PRO MPD Alpha1 powder diffractometer in Bragg–Brentano *θ*/2*θ* geometry of 240 mm of radius with Cu Kα1 radiation (*λ* = 1.5406 Å) at 45 kV and 40 mA, focalizing Ge (111) primary monochromator, with sample spinning at 2 revolutions per s, fixed divergence slit of 0.25º, was used. The measurement range (2*θ*) was from 2º to 50º with a step size of 0.033º and measuring time of 100 s per step. To calculate the CrI of cellulose from the XRD spectra, the peak height method used elsewhere was applied [34].

Total crystallinity index (TCI) was measured using Fourier transform infrared (FTIR) spectra as previously described [35].

Morphological characterization of film surface was performed by field emission scanning electron microscopy (FESEM) (JSM 7100 F) using a LED filter and a backscattered electron detector (BED).

## Results and discussion

The initial cotton linters were long fibres, with an average length of 0.47 mm, which had been hornified (stiffened) during the drying inherent to their production. These traits made them difficult to process because they usually clog in the high-pressure homogenization apparatus. For this reason, they were mechanically refined by beating in a valley mill, which reduced fibre length to 0.25 mm and facilitated their homogenization.

**Fig. 1:**
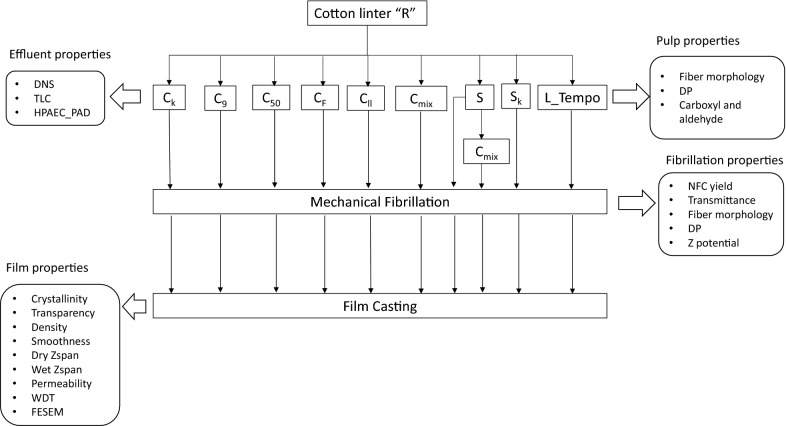
General schema of the experimental work performed

The effect of enzymes on the refined cotton linters was firstly assessed on the properties of effluents released. Then, modifications produced by enzymes in fibre morphology and cellulose were analysed, together with their effect on the fibrillation improvement. Finally, the optical, physical and barrier properties of the films casted from the treated fibres were evaluated (Fig. 1) and compared with films obtained from commercial NFCs.

### Effect of enzymes on sugar release

Cotton linters were treated with different cellulases: endoglucanase Cel9B (C_9_) and commercial cellulases C_50_, C_F_ or C_ll_, and the amount of neutral sugars released was analysed by DNS (Table 1). C_9_ and C_F_ produced a similar sugar release, much lower than that released by C_50_ and C_ll_. When C_F_ and C_ll_ were applied in the same treatment (C_mix_) the sugar release was the same as that with C_ll_ alone.

**Table 1:** Neutral sugar and cellobionic acid release produced by the enzymatic pretreatments

	Sugar release (mg g^−1^ pulp)	Cellobionic acid release (mg g^−1^ pulp)
C_k_	0	0
C_9_	22 ± 4	0
C_50_	90 ± 10	0
C_F_	34 ± 5	0
C_ll_	141 ± 11	0
C_mix_	126 ± 13	0
S	–	0.8 ± 0.2
SC_mix_	242 ± 2	6.1 ± 1.1

C_k_ (cellulase control treatment), C_9_ (Cel9B), C_50_ (Sertec20 cellulase), C_F_ (Fibercare cellulase), C_ll_ (Celluclast cellulase), C_mix_ (cellulase mixture consisting in Fibercare and Celluclast), S (LPMO), SC_mix_ (LPMO and C_mix_)

TLC analysis showed that C_9_ released mainly glucose and cellobiose (Additional file 1), being cellobiose the most abundant cellooligosaccharide released in accordance with its processive endoglucanase activity [27]. Similar product pattern was reported by Garcia-Ubasart et al. [36] when treating flax pulp with this enzyme. Commercial cellulases released a wider pattern of products from cotton linters, neutral sugars from glucose to cellotetraose, without noticeable differences among the enzymes (Additional file 1).

**Fig. 2:**
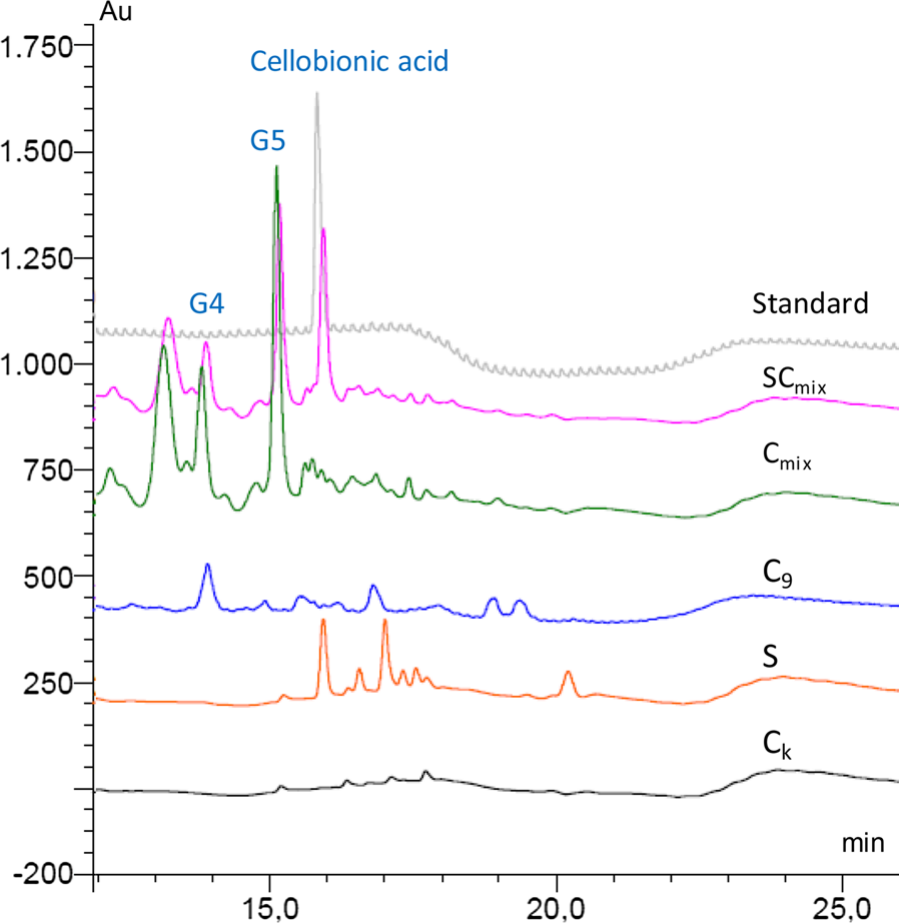
Sugar release produced by C_k_ (control treatment), C_9_ (Cel9B), C_mix_ (cellulase mixture consisting in Fibercare and Celluclast), S (LPMO) and SC_mix_ (LPMO and C_mix_) analysed by HPAEC-PAD. Enzymatic treatments with cellulases were performed at 50 °C, pH 5, for 18 h with 10 U g^−1^ odp of enzyme. (In the case of C_mix_ the enzymatic dose was 20 U g^−1^ odp.) Enzymatic treatment with LPMO (S) was performed at 50 °C, pH 6, for 72 h with 4 mg of enzyme g^−1^ odp in the presence of ascorbic acid and hydrogen peroxide

The action of LPMO, SamLPMO10C (S), was analysed determining the production of oxidized sugars in the effluents by HPAEC-PAD. S treatment released cellobionic acid and other aldonic acid oligosaccharides of higher molecular weight, together with a small fraction of neutral sugars (Fig. 2). Although the amount of cellobionic acid released to the effluents was low (Table 1), the ability of SamLPMO10C to oxidize cotton linters was demonstrated. Our results are in accordance with the production of C1-oxidized oligosaccharides from phosphoric acid-swollen cellulose (PASC) by SamLPMO10C, which was also able to release aldonic acids from flax fibres [17, 29]. On the contrary, in other reported works, the production of aldonic acids when an LPMO belonging to the AA9 family was applied to softwood kraft pulp was not observed [15].

LPMO have extensively been reported to promote the efficiency of cellulases [12], and in our hands, an increase in sugar release by the combined SC_mix_ treatment, that was twice more than in the single treatment with cellulases, C_mix_, confirmed this statement. Furthermore, the cellobionic acid release in the SC_mix_ treatment was eightfold higher than with S, suggesting that the oxidized fractions of cellulose created during S were cleaved and released to the effluent during the C_mix_ treatment (Table 1). C_mix_ treatment was not applied after L_Tempo treatment since some authors state that cellulose oxidation produced by L_Tempo impairs the action of these enzymes [37].

### Effect of enzymes on fibre morphology and cellulose modification

The refined cotton linter fibres used were short (0.25 mm average length) and had a large amount (more than 50%) of fibres lower than 0.2 mm (fines) (Additional file 2a). Enzyme treatment changed the morphology and size distribution of fibres. Cellulases acted on the longer fibres (around 0.2–7.6 mm) creating high amounts of fines, which showed the highest increase in their shortest fraction, fibres lower than 0.1 mm. Among the cellulases, C_9_ produced the smaller morphology change. It slightly reduced fibre length, with only 3% increase in the fine content, and it did not produce a significant effect on fibre width (Table 2). Fibre degradation by C_ll_ was higher (16% fines increase), in accordance with its higher sugar release. Although C_50_ released more quantity of sugars than C_F_, they produced a similar fibre degradation (fines increased in 10%). Combined cellulase treatment (C_mix_) produced the highest increase in fines content (31%), although sugar release was not increased in the combined treatment. Fibre width was slightly reduced by C_ll_ and C_mix_ probably because of the degradation of the surface fibrillation of fibres.

**Table 2:** Effects of enzymatic pretreatments on fibre morphology and on mechanical fibrillation

	Pulp			After mechanical fibrillation	
	Fibre width (μm)	Fibre length (mm)	Fines (%)	Yield (%)	Transmittance at 700 nm (%)
R	25.5	0.25	52.58	0	2.4 ± 0.5
C_k_	24.9	0.25	53.7	0	3.2 ± 0.7
C_9_	24.7	0.24	55.43	0	7.8 ± 0.3
C_50_	24.2	0.22	59.25	0	45.6 ± 1.2
C_F_	24.8	0.22	59.01	0	32.9 ± 0.9
C_ll_	23.2	0.21	62.18	0	27.9 ± 0.8
C_mix_	21.2	0.18	70.44	18 ± 1	52.7 ± 0.9
S_k_	25.1	0.25	52.8	0	3.8 ± 0.5
S	23.3	0.21	61.58	2.5 ± 2	4.3 ± 0.4
SC_mix_	23.2	0.16	73.5	23 ± 1	51.8 ± 0.5
L_Tempo	23.0	0.23	57.13	10 ± 2	4.5 ± 0.6
Com	–	–	–	11 ± 2	5.7 ± 0.7

The confidence interval of the fibre width, fibre length and fines was less than 2% in all cases

R (initial refined pulp), C_k_ (cellulase control treatment), C_50_ (Sertec20 cellulase), C_F_ (Fibercare cellulase), C_ll_ (Celluclast cellulase) C_9_ (Cel9B), C_mix_ (cellulase mixture consisting in Fibercare and Celluclast), S_k_ (LPMO control treatment), S (LPMO), SC_mix_ (LPMO and C_mix_), L_Tempo (Laccase_Tempo treatment) and Com (commercial NFC)

Whereas hydrolytic treatments with cellulases are well known to act on fibre morphology [38], little knowledge exists about the fibre modification produced by oxidative treatments, particularly with LPMO enzymes. Interestingly, the two oxidative treatments performed affected fibre morphology, reducing its fibre length and width and consequently increasing fines content (Table 2). The increase in fines content was more pronounced with S (16%) than with L_Tempo (6%). These results contrast with those reported by Aracri et al. [8] reporting that no effect on fines content was produced by L_Tempo treatment of sisal pulps. Finally, SC_mix_ treatment produced the highest increase in fines (37%) and a large amount of fines lower than 0.1 mm (42%) (Additional file 2b), in agreement with the highest sugar release of SC_mix_ treatment, confirming that fibre degradation by cellulases was boosted by LPMO action. These results are in accordance with the proposed mechanism of LPMO that create nicking points where the cohesion of the fibres was decreased, improving the attack of cellulases [15].

**Fig. 3:**
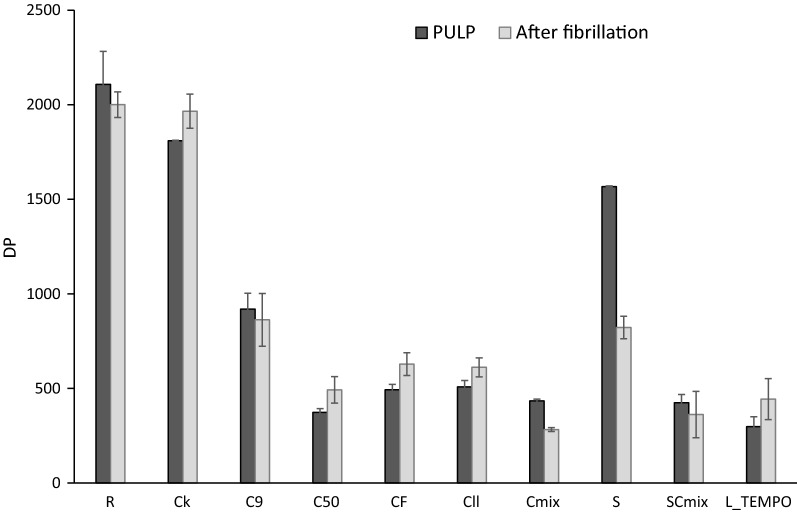
Effect of enzymatic treatments on cellulose degree of polymerization. R (initial refined pulp), C_k_ (control treatment), C_9_ (Cel9B), C_50_ (Sertec20 cellulase), C_F_ (Fibercare cellulase), C_ll_ (Celluclast cellulase), C_mix_ (cellulase mixture consisting in Fibercare and Celluclast), S (LPMO), SC_mix_ (LPMO and C_mix_) and L_Tempo (Laccase_Tempo treatment). Enzymatic treatments with cellulases were performed at 50 °C, pH 5, for 18 h with 10 U g^−1^ odp of enzyme. (In the case of C_mix_ the enzymatic dose was 20 U g^−1^.) Enzymatic treatment with LPMO (S) was performed at 50 °C, pH 6, for 72 h with 4 mg of enzyme g^−1^ odp in the presence of ascorbic acid and hydrogen peroxide. Enzymatic treatment with Laccase_Tempo was performed at room temperature, pH 5, for 18 h at 60 U g^−1^ odp of laccase and 8% odp of Tempo

Changes in cellulose polymerization were assessed via intrinsic viscosity measurements (Fig. 3). Similarly to what has been reported [26, 39] all the tested cellulases decreased DP. In correlation with the effects of the cellulases on fibre morphology and sugar release described above, C_9_ produced lower cellulose degradation (52% decrease in DP) than the commercial cellulases applied (around 73–79%). A similar cellulose depolymerization was observed by Qing et al. in 2013 [24] when C_F_ and C_ll_ were applied to a bleached eucalyptus kraft pulp at lower enzymatic doses. Contrary to our results, previous authors reported that DP of softwood and flax pulps was not significantly affected by endoglucanase C_9_ [36, 38], but in our case, the higher cellulose degradation produced by C_9_ in cotton linters can be due to the longer treatment applied (18 h vs. 1–2 h in previous works).

Concerning the oxidative treatments, cellulose DP was highly affected by L_Tempo, while S treatment produced a small decrease (Fig. 3). Depolymerization of cellulose by L_Tempo has been described to be produced by active species such as hydroxyl radicals formed in situ by side reactions of the hydroxylamine structure with oxygen during the oxidative treatment [40]. Moreover, the presence of aldehyde groups produced by L_Tempo treatment can provide underestimation of viscosity values. These aldehyde groups can give depolymerization reactions through β-elimination during the viscosity determination method, with cupriethylenediamine at alkaline conditions. To avoid this problem, viscosity was also measured after treating the pulp samples with sodium borohydride (borohydride viscosity) in order to inactivate carbonyl groups by reduction to hydroxyl groups [8]. Borohydride viscosity was measured in all the samples (data not shown) obtaining DP values similar to those shown in Fig. 3, with the exception of L_Tempo sample that showed an increased DP, indicating that aldehyde groups were formed in this treatment. However, even after the reductive treatment, DP of L_Tempo sample was low (572), indicating degradation of cotton linters cellulose by L_Tempo, similarly to previous results described for sisal pulps, although with less intense degradation [8]. On the contrary, the low depolymerization produced by LPMO suggested that this enzyme affects fibre morphology without significantly degrading cellulose. Villares et al. also reported a slight decrease in DP by LPMO although fibre morphology was not affected [15]. Interestingly, despite the high fibre modification and cellobionic acid release by the S treatment, cellulose was not significantly degraded. Subsequent treatment with the cellulase mixture, SC_mix_, did not increase cellulose depolymerization by cellulases.

Finally, the creation of functional groups on cellulose was evaluated by measuring the carboxyl and aldehyde content of fibres. Results showed a significant increase of these groups only with the oxidative treatments, where the L_Tempo treated pulps exhibited the highest content (Table 3), as also appreciated by FTIR spectra (Additional file 3). A different mechanism of creating COOH groups was produced among the oxidative treatments: whereas L_Tempo oxidized cellulose as a result of the conversion of C6 primary hydroxyl groups in cellulose via an aldehyde group [41], S created COOH through oxidation of alcohol in C1 position [29]. A small fraction of aldehydes were also produced during L_Tempo in accordance with the previous results on borohydride viscosity. The modest increase in carboxyl group content provided by the L_Tempo system in comparison with other works under the conditions used is probably due to the absence of added oxygen during the treatment [7].

**Table 3:** Effects of enzymatic pretreatments in the creation of COOH and CHO groups in cellulose fibres

	COOH (µmol g^−1^ odp)	CHO (µmol g^−1^ odp)
R	18 ± 4	0
C_k_	13 ± 1	0
C_9_	20 ± 1	1 ± 1
C_mix_	17 ± 3	1 ± 1
S	29 ± 4	0
SC_mix_	20 ± 1	0
L_Tempo	85 ± 9	4 ± 1

R (initial refined pulp), C_k_ (cellulase control treatment), C_9_ (Cel9B), C_mix_ (cellulase mixture consisting in Fibercare and Celluclast), S (LPMO), SC_mix_ (LPMO and C_mix_) and L_Tempo (Laccase_Tempo treatment)

The oxidative cleavage of cellulose by S treatment leads to the formation of a small quantity of aldonic acids (COOH groups) at the C1 position. However, a fraction of these COOH groups disappeared when C_mix_ was applied after S, suggesting they were removed. This result is in accordance with the higher cellobionic acid release in the SC_mix_ treatment previously shown, corroborating that the oxidized fractions of cellulose created by S activity were cleaved by the C_mix_ treatment.

### Effect of enzymes on fibrillation improvement

Enzyme-treated samples were homogenized at high pressure, and their properties were analysed. NFC was only obtained in the hydrolytic treatment with the cellulase mixture C_mix_ (simultaneous application of C_F_ and C_ll_), while none of the other cellulase treatments gave significant amount of NFC (Table 2). Nanofibrillation of this sample may have been promoted by its low fibre length (70% of fines) or by the decrease of hornification produced by these cellulases [19, 42]. A lower yield of NFC was obtained with oxidative S and L_Tempo treatments. In these treatments, nanofibrillation was probably stimulated by the presence of COOH groups, as it has been reported [43, 44]. The highest yield of NFC (23%) was produced with the SC_mix_ pretreatment, in concordance with the best performance observed in the other parameters evaluated, where a higher effect of cellulases after an LPMO treatment was achieved. This yield increase produced by LPMO can be related to the introduction of nicks in the most crystalline regions of cellulose molecules (as suggested by Villares et al. and Valenzuela et al. [15, 17]), rather than to the small increase of COOH that are left on fibres after cellulase treatment. Recently, it has been reported the nanofibrillation of flax pulp after a sequential pretreatment of SamLPMO10C and C_9_, obtaining a similar yield of 24% [17]. Remarkably, the NFC yield obtained with C_mix_ and SC_mix_ was higher than the NFC content of a commercial nanocellulose (Table 2).

Despite the fact that in some samples no NFC was obtained, other parameters were measured in order to analyse the fibrillation improvements produced by the enzymes. For example, transmittance is a simple mean to get an approximate idea about the width of the ensuing fibrils. In fact, when light passes through a medium containing randomly dispersed particles, it is scattered by the particles causing a reduction in the transparency degree, as previously reported [24]. Transmittance at 700 nm was strongly improved by hydrolytic treatments in all the samples (Table 2), suggesting a decrease in the amount of non-fibrillated and partially fibrillated fractions responsible for light scattering phenomenon. In accordance with the NFC yield results, the highest improvement in transmittance was produced with C_mix_ and SC_mix_ samples. Although fibres of lower length were created with C_ll_ treatment, a higher transmittance value was obtained with C_50_, followed by C_F_, C_ll_ and C_9_. Concerning the oxidative treatments, they only improved transmittance to less than 5%. The higher carboxyl content of L_Tempo sample did not produce a significant increase in transmittance, in accordance with the observations of Besbes et al., 2011, who reported that COOH content has to be higher than 300 μmol g^−1^ odp to produce a significant increase in transmittance [44].

Zeta potential is a measure of the magnitude of the electrostatic or charge repulsion/attraction between particles and is one of the fundamental parameters known to affect stability. All the samples obtained after mechanical fibrillation had a Z potential around − 30 mV, that indicates that there is no agglomeration, which means sufficient mutual repulsion resulting in colloidal stability. This value was slightly increased with the oxidative treatments to − 40 mV probably due to the COOH groups (Additional file 4). However, it was reduced in the SC_mix_ treatments, correlating again the removal of the LPMO-produced COOH groups by the cellulase treatment. A similar result has been reported in NFC from flax and bleached kraft pulp [16, 17].

Although it has been reported that DP can be reduced during fibrillation [24, 25, 45], in our results DP was not affected after the pass through the high-pressure homogenizer (Fig. 3). In fact, only in the S sample the DP decreased. Maybe the oxidation of glycosidic linkages during the treatment with LPMO rendered cellulose more susceptible to be degraded during fibrillation. Finally, it has to be pointed out the low DP of C_mix_ and SC_mix_ samples, indicating that cellulose chains were only formed by ≈ 300 glucose units. This value was only slightly higher than in cellulose nanocrystals (≈ 200 glucose units) obtained from cotton linters [35].

### Effect of enzymes on film properties

Films of ~ 45 μm thickness were prepared, and their optical, physico-mechanical and barrier properties were measured (Table 4). Crystallinity of films was determined by XRD. It was high in all the samples (around 90%), as expected for cotton linters, although they had suffered multiple passes through the homogenizer, a process that has been reported to reduce crystallinity [46]. Values obtained are similar to that reported by Hideno et al. in 2016 and higher to that obtained by Saito et al. in 2006 [19, 47]. Cellulases treatment slightly increased crystallinity of films, probably due to their action on the amorphous zones of cellulose more susceptible to be attacked by these enzymes [48], a phenomenon observed also when commercial cellulases were applied to bleached wood pulps [24, 26]. Crystallinity is also an important parameter that affects the action of LPMO enzymes, where, on the contrary, higher crystalline cellulose seems to be a better substrate to be oxidized [17, 49]. Interestingly, in our experiments, this property was not negatively affected by the S treatment, similarly to what has been reported for NFC from flax pulps [17]. The other oxidative treatment, L_Tempo, did not affect to this property neither, as previously reported [47]. The lower DP produced with the enzymatic treatments did not affect cellulose crystallinity. This property was also measured from the FTIR spectra obtaining the total crystallinity index (TCI) (Additional file 3). It had a value around 1.2, without significant differences between samples, in accordance with values obtained by XRD.

**Table 4:** Effects of enzymatic pretreatments in crystallinity and physical properties of the films obtained after mechanical fibrillation

	Crystallinity (%)	Transparency (%)		Density (g cm^−3^)	Bekk smoothness (s)
		Upper face	Lower face		Upper face
R	89.1 ± 0.1	27.8 ± 0.9	28.0 ± 0.1	0.8 ± 0.1	15 ± 4
C_k_	88.5 ± 0.5	35.9 ± 3.7	36.0 ± 3.7	0.8 ± 0.1	10 ± 1
C_9_	90.3 ± 0.2	54.3 ± 0.4	52.9 ± 0.2	1.0 ± 0.1	29 ± 5
C_50_	91.4 ± 0.1	83.4 ± 0.5	83.2 ± 1.1	1.1 ± 0.0	46 ± 6
C_F_	91.0 ± 0.3	74.9 ± 0.7	73.8 ± 0.5	1.1 ± 0.1	53 ± 2
C_ll_	91.4 ± 0.2	75.7 ± 0.9	74.7 ± 0.8	1.1 ± 0.0	34 ± 2
C_mix_	92.4 ± 0.2	86.1 ± 0.5	85.9 ± 1.5	0.9 ± 0.0	50 ± 6
S_k_	89.0 ± 0.2	52.9 ± 1.8	51.5 ± 2.3	0.8 ± 0.1	11 ± 2
S	90.5 ± 0.5	60.0 ± 0.4	60.9 ± 0.3	0.9 ± 0.0	10 ± 2
SC_mix_	92.5 ± 0.1	85.6 ± 0.5	85.3 ± 0.5	1.1 ± 0.1	53 ± 1
L_Tempo	89.8 ± 0.1	38.9 ± 3.2	37.8 ± 1.8	0.9 ± 0.1	14 ± 3
Com	81.1 ± 0.2	66.2 ± 0.7	66.7 ± 0.4	1.0 ± 0.0	19 ± 3

R (initial refined pulp), C_k_ (cellulase control treatment), C_9_ (Cel9B), C_50_ (Sertec20 cellulase), C_F_ (Fibercare cellulase), C_ll_ (Celluclast cellulase), C_mix_ (cellulase mixture consisting in Fibercare and Celluclast), S_k_ (LPMO control treatment), S (LPMO), SC_mix_ (LPMO and C_mix_), L_Tempo (Laccase_Tempo treatment) and Com (commercial NFC)

Transparency of films was determined, showing agreement with transmittance of homogenized suspensions previously shown, and no significant differences were found between upper and lower faces of films (Table 4). The highest transparency was achieved with C_mix_ and SC_mix_, where the increase in transparency was around 50 points (see Additional file 5). C_50_, C_F_, C_ll_ and C_9_ increased this property in 47, 39, 39 and 18 points, respectively. Transparency obtained with C_mix_ treatments was similar to that reported by Hideno et al. in 2016 with cotton linters and cellulase, and also to Chen et al. in 2014 in a NFC/acrylic resin composite sheet [19, 21]. S showed a lower increase in transparency of 7 points, being these films of higher transparency than L_Tempo films. Interestingly, crystallinity and transparency of films from cellulase treatments were higher than those of the films made from commercial NFC.

The density of the films obtained (Table 4) was comparable to that of films obtained from bacterial cellulose and considerably higher than papers from wood fibres [50]. Enzymatic treatment with cellulases produced the films of higher density probably due to the lower fibre size. Smoothness of non-treated films (R) was similar to films obtained from commercial NFC (Table 4). Whereas smoothness was not affected by the oxidative treatments, this property was significantly increased with all the cellulases applied, particularly with C_50_, C_F_ and C_mix_ treatments. No differences were appreciated if C_mix_ was applied after S. Increased values of smoothness were obtained in the lower face of films (data not shown). The high values of smoothness and transparency of the obtained films give them the potential to be applied for printed electronics [51].

**Fig. 4:**
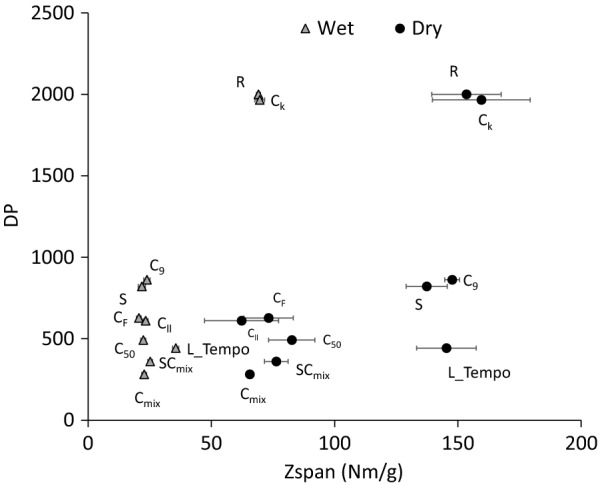
DP of cellulose *vs.* mechanical resistance of NFC films. R (initial refined pulp), C_k_ (control treatment), C_9_ (Cel9B), C_50_ (Sertec20 cellulase), C_F_ (Fibercare cellulase), C_ll_ (Celluclast cellulase), C_mix_ (cellulase mixture consisting in Fibercare and Celluclast), S (LPMO), SC_mix_ (LPMO and C_mix_) and L_Tempo (Laccase_Tempo treatment). Enzymatic treatments with cellulases were performed at 50 °C, pH 5, for 18 h with 10 U g^−1^ odp of enzyme. (In the case of C_mix_ the enzymatic dose was 20 U g^−1^.) Enzymatic treatment with LPMO (S) was performed at 50 °C, pH 6, for 72 h with 4 mg of enzyme g^−1^ odp in the presence of ascorbic acid and hydrogen peroxide. Enzymatic treatment with Laccase_Tempo was performed at room temperature, pH 5, for 18 h at 60 U g^−1^ odp of laccase and 8% odp of Tempo

Mechanical resistance of films was determined (Fig. 4). Non-treated films (R) showed dry zero-span index of 153 Nm g^−1^, similar to that of commercial NFC films (185 Nm g^−1^) and higher than that of the paper from unbleached kraft pulp reinforced with cotton linters NFC [52] (8 Nm g^−1^). As previously said, DP was affected by the action of enzymes, and consequently, this could affect the physical properties of the resulting films. Interestingly, although cellulose was partially degraded with all the treatments, dry zero-span index of C_9_, S and L_Tempo samples was not significantly affected, while a significant reduction in this property was produced with all the commercial cellulases used (Fig. 4). In order to evaluate the resistance offered by a single fibre, wet zero-span index was also measured. The resistance in all the samples was reduced around 55–84%, including the one from commercial NFC. Whereas non-treated films had values around 70 Nm g^−1^, this value was reduced to 25 Nm g^−1^ in all the enzymatic treated samples, without significant differences between them. Films from commercial NFC had a slightly higher wet zero-span index (42 Nm g^−1^). These wet zero-span values obtained were significantly lower than those reported for bacterial cellulose films (around 100 Nm g^−1^) [50], probably as a result of the higher crystallinity of bacterial nanocellulose.

**Fig. 5:**
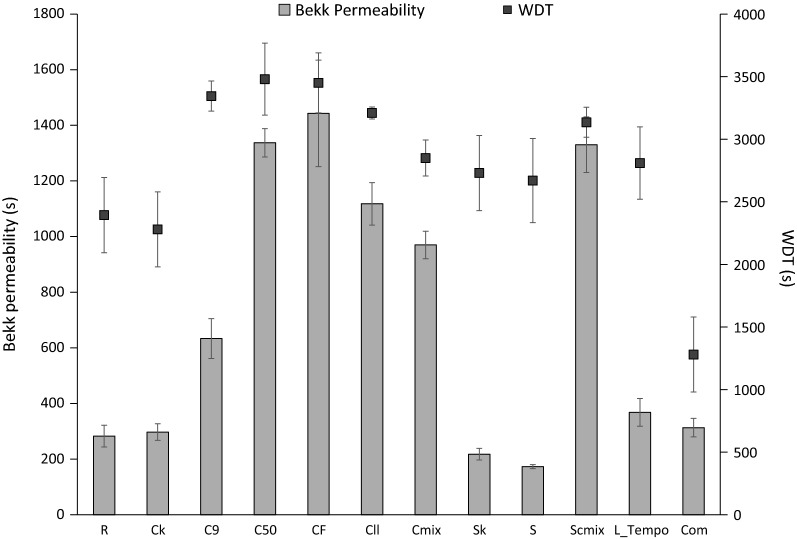
Barrier properties to air (Bekk permeability) and water (water drop test) of NFC films. R (initial refined pulp), C_k_ (cellulase control treatment), C_9_ (Cel9B), C_50_ (Sertec20 cellulase), C_F_ (Fibercare cellulase), C_ll_ (Celluclast cellulase), C_mix_ (cellulase mixture consisting in Fibercare and Celluclast), S_k_ (LPMO control treatment), S (LPMO), SC_mix_ (LPMO and C_mix_), L_Tempo (Laccase_Tempo treatment) and Com (commercial NFC). Enzymatic treatments with cellulases were performed at 50 °C, pH 5, for 18 h with 10 U g^−1^ odp of enzyme. (In the case of C_mix_ the enzymatic dose was 20 U g^−1^.) Enzymatic treatment with LPMO (S) was performed at 50 °C, pH 6, for 72 h with 4 mg of enzyme g^−1^ odp in the presence of ascorbic acid and hydrogen peroxide. Enzymatic treatment with Laccase_Tempo was performed at room temperature, pH 5, for 18 h at 60 U g^−1^ odp of laccase and 8% odp of Tempo

Barrier properties to air and water of the obtained films were also measured. Air permeability was measured by the Bekk method (Fig. 5). The non-treated sample (R) had a similar value than films from commercial NFC. Interestingly, cellulases strongly increased the seconds that the air needed to pass through the films, i.e. decreased permeability. The most notable effect was produced with C_F_, C_50_ and SC_mix_ followed by C_ll_, C_mix_ and C_9_. On the other hand, oxidative treatments did not produce significant effects. Permeability of films from cellulase-treated samples was threefold higher than that of commercial NFC films, indicating that a strongly closed structure was formed after the enzymatic treatments. The increased fine content and fibrillation obtained with the cellulase treatments are consistent with an increased cohesion between fibre surfaces and responsible for the decreased paper permeability. These results are consistent with those of Cadena et al. who found cellulase treatments to reduce the air permeance of paper [53]. Similar to smoothness, permeability was strongly decreased in the lower face of the film (data not shown).

Film permeability was intensely related to the barrier property to water, measured by the WDT (Fig. 5). All the films from enzyme-treated samples showed an increased water impermeability although the effect was more noticeable with cellulase treatments, which showed a maximum value of 3150 s in C_F_ sample. In spite of the high impermeability compared to current cellulosic papers, it was lower than that provided by bacterial cellulose films (4000 s) [50]. Interestingly, non-treated films had lower permeability to water than commercial NFC films, maybe because of the higher crystallinity of the cotton linters used.

**Fig. 6:**
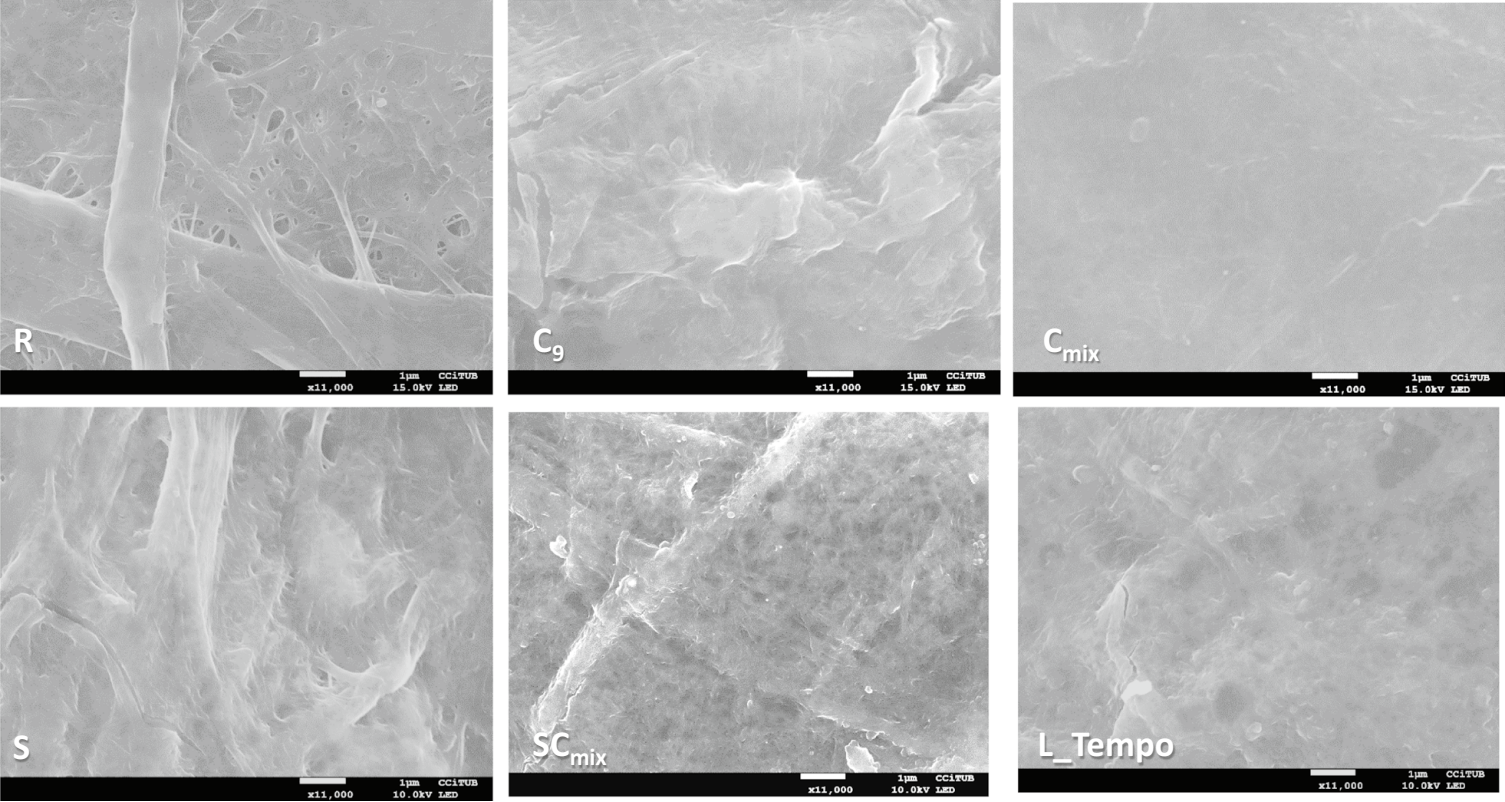
FESEM images of NFC films from non-treated and enzymatically treated samples. R (initial refined pulp), C_9_ (Cel9B), C_mix_ (cellulase mixture consisting in Fibercare and Celluclast), S (LPMO), SC_mix_ (LPMO and C_mix_) and L_Tempo (Laccase_Tempo treatment). Enzymatic treatments with cellulases were performed at 50 °C, pH 5, for 18 h with 10 U g^−1^ odp of enzyme. (In the case of C_mix_ the enzymatic dose was 20 U g^−1^.) Enzymatic treatment with LPMO (S) was performed at 50 °C, pH 6, for 72 h with 4 mg of enzyme g^−1^ odp in the presence of ascorbic acid and hydrogen peroxide. Enzymatic treatment with Laccase_Tempo was performed at room temperature, pH 5, for 18 h at 60 U g^−1^ odp of laccase and 8% odp of Tempo

Finally, film surface morphology was analysed by FESEM (Fig. 6). Non-treated films (R) showed fibres of different lengths and fibre widths with fibrillation. A highly entangled nano- and/or micro-fibre network was observed in enzyme-treated samples, similarly to that reported by Hu et al. in 2018 and Tarrés et al. in 2017 [16, 39]. Surface morphology of films demonstrates that the enzymatic treatments performed boosted mechanical delamination, since those films showed a compact structure and their structure was difficult to be visualized. Moreover, in SC_mix_ films a thin layer of nanofibres surrounding bigger fibres was appreciated.

Although the presence of NFC material was not detected in films from individual cellulases and oxidative enzymes, the film properties obtained clearly show that these treatments improved fibrillation. Moreover, according to the optical, physical and barrier properties obtained, the films from enzymatically treated cotton linters seem very promising to obtain biomaterials that could replace petrol-based products.

## Conclusions

Four hydrolytic enzymes (cellulases) were applied on cotton linters, affecting fibre morphology and degrading cellulose differently. Improved mechanical fibrillation and 18% NFC yield were obtained with a cellulase mixture (C_mix_). Application of oxidative enzymes (LPMO and L_Tempo) introduced COOH groups into cellulose. The amount of COOH groups created with L_Tempo allowed the production of NFC during mechanical fibrillation (10%). However, the smaller quantity of these groups introduced by LPMO was not enough to produce NFC. The main difference between the two oxidative treatments was that L_Tempo degraded cellulose, whereas LPMO had more effect on fibre degradation. LPMO (S) boosted the action of cellulases although the COOH groups created were released to effluents after the hydrolytic treatment. Films with high crystallinity (92%) and transparency (86%), increased smoothness, and high air and water barrier properties were obtained after cellulase treatment and mechanical fibrillation on cotton linters. The introduction of an LPMO treatment before cellulase mixture (SC_mix_ treatment) produced higher NFC yield (23%) without a further improvement in film properties.

## Additional files

**Additional file 1.** TLC analysis of sugars released during enzymatic treatments performed at 50 °C, pH 5, during 18 h with 10 U g^−1^ odp of enzyme (in the case of Cmix the enzymatic dose was 20 U g^−1^ odp). C_9_ (Cel9B), C_50_ (Sertec20 cellulase), C_F_ (Fibercare cellulase), C_ll_ (Celluclast cellulase), C_mix_ (cellulase mixture consisting in Fibercare and Celluclast). M) size markers of glucose (G), cellobiose (G2), cellotriose (G3), cellotetraose (G4) and cellopentaose (G5).

**Additional file 2.** Effect of the hydrolytic (a) and oxidative and C_mix_ (b) enzymatic pretreatments on fibre length distribution. The confidence interval of the length fibre distribution was less than 2% in all cases.

**Additional file 3.** FTIR spectra of obtained films from control treatment (C_k_) and Laccase_Tempo treatment (L_Tempo). TCI was calculated from the ratio of the absorptions at 1372 and 2900 cm^−1^. The peak detected at 1750 cm^−1^ with L_Tempo correspond to the COOH groups.

**Additional file 4.** Z potential values of the samples obtained after mechanical fibrillation. R (initial refined pulp), C_k_ (control treatment), C_9_ (Cel9B), C_mix_ (cellulase mixture consisting in Fibercare and Celluclast), S (LPMO), SC_mix_ (LPMO and C_mix_) and L_Tempo (Laccase_Tempo treatment).

**Additional file 5.** Obtained films from cellulase control C_k_ (a) and C_mix_ (b) pretreatments after mechanical fibrillation.

## Data Availability

The data sets used and analysed during the current study are available from the corresponding author on reasonable request.

## Abbreviations

NFC: nanofibrillated cellulose
R: refined cotton linter
C_k_: cellulase control treatment
LPMO: lytic polysaccharide monooxygenases
S: treatment with LPMO enzyme
S_k_: LPMO control treatment
C9: treatment with endoglucanase Cel9B
C_ll_: treatment with commercial cellulase Celluclast
C_F_: treatment with commercial cellulase Fibercare
C_50_: treatment with commercial cellulase from Sertec20
C_mix_: combined treatment with Celluclast and Fibercare
SC_mix_: treatment with LPMO enzyme followed by C_mix_
L_Tempo: treatment with laccase and Tempo
Com: commercial NFC
DNS: dinitrosalicylic
TLC: thin-layer chromatography
HPAEC-PAD: high-performance anion-exchange chromatography with pulsed amperometric detection
DP: degree of polymerization
WDT: water drop test
FESEM: field emission scanning electron microscopy
Odp: oven-dried pulp
XRD: X-ray powder diffraction
