# Supplementary material for: Assessing the enzymatic effects of cellulases and LPMO in improving mechanical fibrillation of cotton linters
Source: Biotechnol Biofuels. 2019 Jun 26;12:161. doi: 10.1186/s13068-019-1502-z (PMC6593493; doi:10.1186/s13068-019-1502-z)
Supplement: Supplementary file 1 — Additional file 1. TLC analysis of sugars released during enzymatic treatments performed at 50 °C, pH 5, during 18 h with 10 U g−1 odp of enzyme (in the case of Cmix the enzymatic dose was 20 U g−1 odp). C9 (Cel9B), C50 (Sertec20 cellulase), CF (Fibercare cellulase), Cll (Celluclast cellulase), Cmix (cellulase mixture consisting in Fibercare and Celluclast). M) size markers of glucose (G), cellobiose (G2), cellotriose (G3), cellotetraose (G4) and cellopentaose (G5). [file 13068_2019_1502_MOESM1_ESM.docx]

**Additional files**

**Assessing the enzymatic effects of cellulases and LPMO in improving mechanical fibrillation of cotton linters**

Cristina Valls^1,2^ *, F. I .Javier Pastor^1^, M. Blanca Roncero^2^, Teresa Vidal^2^, Pilar Diaz^1^, Josefina Martínez^1^, Susana V. Valenzuela^1*^

^1^ Department of Genetics, Microbiology and Statistics. Faculty of Biology. Universitat de Barcelona. Av. Diagonal 643, 08028 Barcelona, Spain

^2^ CELBIOTECH_Paper Engineering Research Group. Universitat Politècnica de Catalunya, BarcelonaTech, 08222 Terrassa, Spain.

*Correspondence to Cristina Valls cristina.valls[@upc.edu](mailto:tvidal@etp.upc.edu) and Susana V. Valenzuela [susanavalenzuela@ub.edu](mailto:susanavalenzuela@ub.edu)

Additional file 1. TLC analysis of sugars released during enzymatic treatments performed at 50ºC, pH 5, during 18h with 10 U g-1 odp of enzyme (in the case of Cmix the enzymatic dose was 20 U g-1 odp). C_9_ (Cel9B), C_50_ (Sertec20 cellulase), C_F_ (Fibercare cellulase), C_ll_ (Celluclast cellulase), C_mix_ (cellulase mixture consisting in Fibercare and Celluclast). M) size markers of gluccose (G), cellobiose (G2), cellotriose (G3), cellotetraose (G4) and cellopentaose (G5).
