# Supplementary material for: Assessing the enzymatic effects of cellulases and LPMO in improving mechanical fibrillation of cotton linters
Source: Biotechnol Biofuels. 2019 Jun 26;12:161. doi: 10.1186/s13068-019-1502-z (PMC6593493; doi:10.1186/s13068-019-1502-z)
Supplement: Supplementary file 2 — Additional file 2. Effect of the hydrolytic (a) and oxidative and Cmix (b) enzymatic pretreatments on fibre length distribution. The confidence interval of the length fibre distribution was less than 2% in all cases. [file 13068_2019_1502_MOESM2_ESM.docx]

Additional file 2. Effect of the hydrolytic (a) and oxidative and C_mix_ (b) enzymatic pretreatments on fibre length distribution. The confidence interval of the length fibre distribution was less than 2% in all cases
