# Supplementary material for: Assessing the enzymatic effects of cellulases and LPMO in improving mechanical fibrillation of cotton linters
Source: Biotechnol Biofuels. 2019 Jun 26;12:161. doi: 10.1186/s13068-019-1502-z (PMC6593493; doi:10.1186/s13068-019-1502-z)
Supplement: Supplementary file 3 — Additional file 3. FTIR spectra of obtained films from control treatment (Ck) and Laccase_Tempo treatment (L_Tempo). TCI was calculated from the ratio of the absorptions at 1372 and 2900 cm−1. The peak detected at 1750 cm−1 with L_Tempo correspond to the COOH groups. [file 13068_2019_1502_MOESM3_ESM.docx]

Additional file 3. FTIR spectra of obtained films from control treatment (C_k_) and Laccase_Tempo treatment (L_Tempo). TCI was calculated from the ratio of the absorptions at 1372 and 2900 cm^-1^. The peak detected at 1750 cm^-1^ with L_Tempo correspond to the COOH groups.
