# Supplementary material for: Assessing the enzymatic effects of cellulases and LPMO in improving mechanical fibrillation of cotton linters
Source: Biotechnol Biofuels. 2019 Jun 26;12:161. doi: 10.1186/s13068-019-1502-z (PMC6593493; doi:10.1186/s13068-019-1502-z)
Supplement: Supplementary file 4 — Additional file 4. Z potential values of the samples obtained after mechanical fibrillation. R (initial refined pulp), Ck (control treatment), C9 (Cel9B), Cmix (cellulase mixture consisting in Fibercare and Celluclast), S (LPMO), SCmix (LPMO and Cmix) and L_Tempo (Laccase_Tempo treatment). [file 13068_2019_1502_MOESM4_ESM.docx]

Additional file 4. Z potential values of the samples obtained after mechanical fibrillation. R (initial refined pulp), C_k_ (control treatment), C_9_ (Cel9B), C_mix_ (cellulase mixture consisting in Fibercare and Celluclast), S (LPMO), SC_mix_ (LPMO and C_mix_) and L_Tempo (Laccase_Tempo treatment).
