# Supplementary material for: Assessing the enzymatic effects of cellulases and LPMO in improving mechanical fibrillation of cotton linters
Source: Biotechnol Biofuels. 2019 Jun 26;12:161. doi: 10.1186/s13068-019-1502-z (PMC6593493; doi:10.1186/s13068-019-1502-z)
Supplement: Supplementary file 5 — Additional file 5. Obtained films from cellulase control Ck (a) and Cmix (b) pretreatments after mechanical fibrillation. [file 13068_2019_1502_MOESM5_ESM.docx]

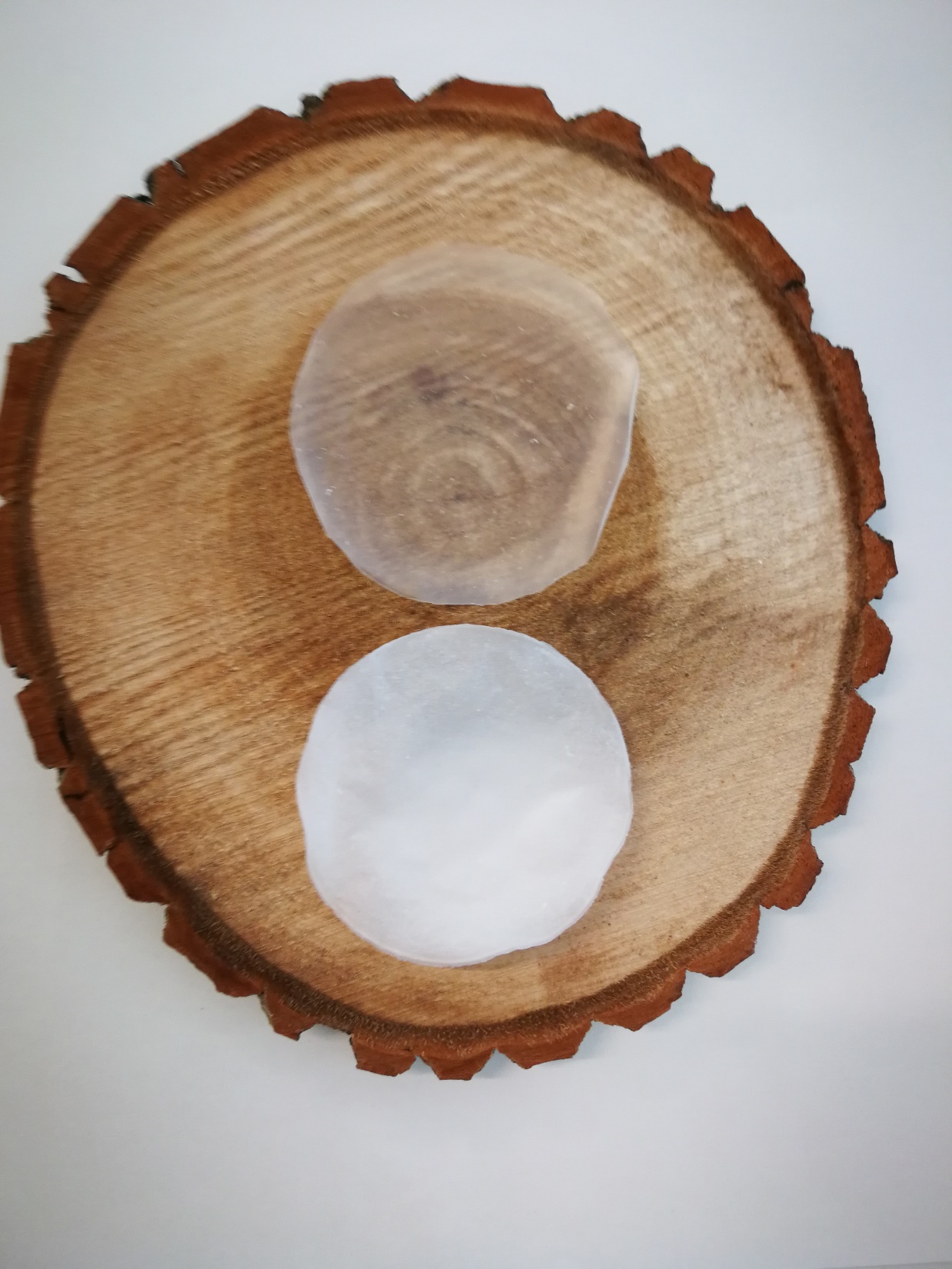

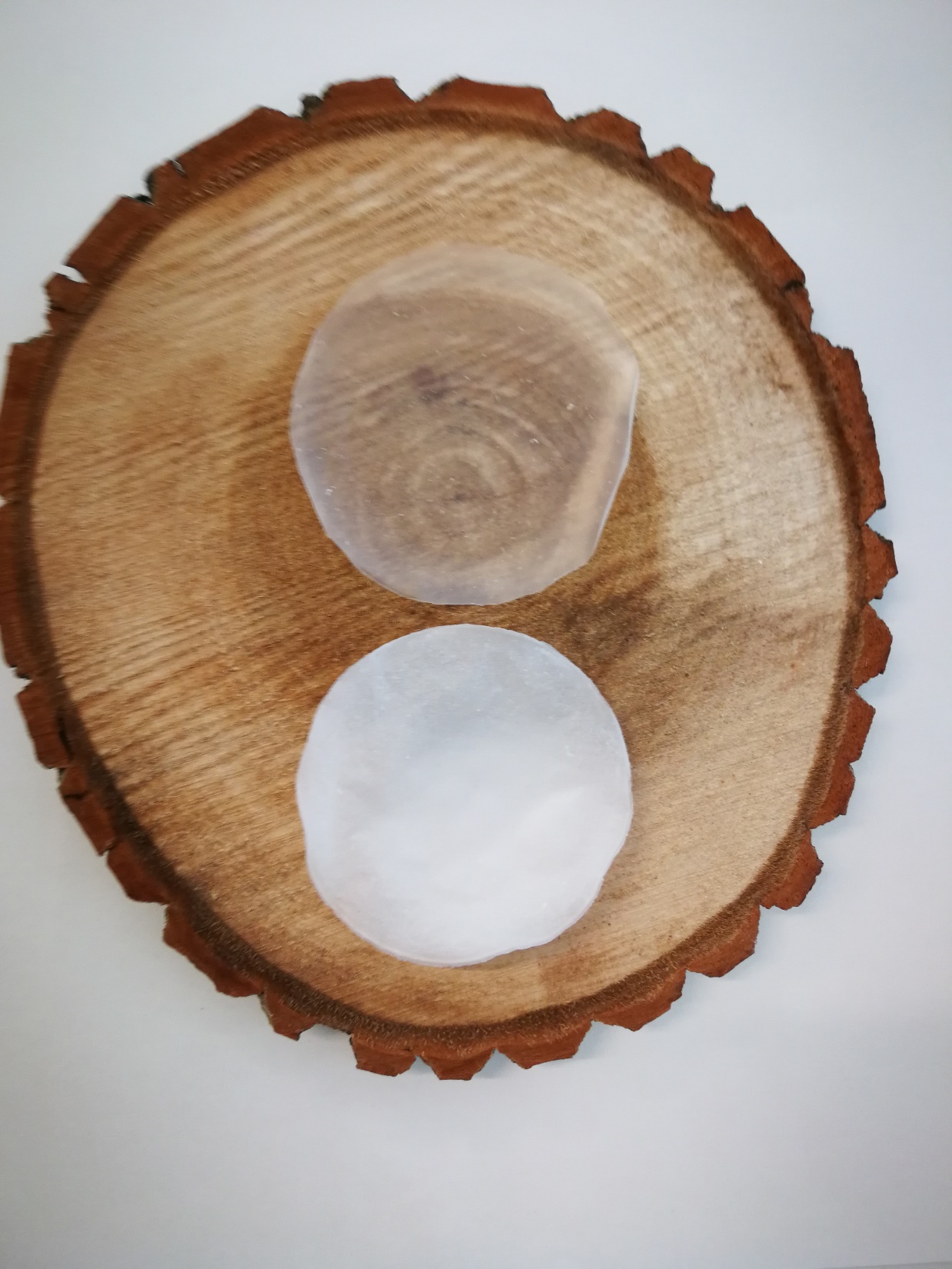


Additional file 5. Obtained films from cellulase control C_k_ (a) and C_mix_ (b) pretreatments after mechanical fibrillation
